# Apparent Diffusion Coefficient Map–Based Radiomics Features for Differential Diagnosis of Pleomorphic Adenomas and Warthin Tumors From Malignant Tumors

**DOI:** 10.3389/fonc.2022.830496

**Published:** 2022-06-07

**Authors:** Baohong Wen, Zanxia Zhang, Jing Zhu, Liang Liu, Yinhua Li, Haoyu Huang, Yong Zhang, Jingliang Cheng

**Affiliations:** ^1^ Department of MRI, the First Affiliated Hospital of Zhengzhou University, Zhengzhou, China; ^2^ Advanced Technical Support, Philips Healthcare, Shanghai, China

**Keywords:** radiomics, diffusion-weighted image, apparent diffusion coefficient, parotid gland tumor, magnetic resonance imaging

## Abstract

**Purpose:**

The magnetic resonance imaging (MRI) findings may overlap due to the complex content of parotid gland tumors and the differentiation level of malignant tumor (MT); consequently, patients may undergo diagnostic lobectomy. This study assessed whether radiomics features could noninvasively stratify parotid gland tumors accurately based on apparent diffusion coefficient (ADC) maps.

**Methods:**

This study examined diffusion-weighted imaging (DWI) obtained with echo planar imaging sequences. Eighty-eight benign tumors (BTs) [54 pleomorphic adenomas (PAs) and 34 Warthin tumors (WTs)] and 42 MTs of the parotid gland were enrolled. Each case was randomly divided into training and testing cohorts at a ratio of 7:3 and then was compared with each other, respectively. ADC maps were digitally transferred to ITK SNAP (www.itksnap.org). The region of interest (ROI) was manually drawn around the whole tumor margin on each slice of ADC maps. After feature extraction, the Synthetic Minority Oversampling TEchnique (SMOTE) was used to remove the unbalance of the training dataset. Then, we applied the normalization process to the feature matrix. To reduce the similarity of each feature pair, we calculated the Pearson correlation coefficient (PCC) value of each feature pair and eliminated one of them if the PCC value was larger than 0.95. Then, recursive feature elimination (RFE) was used to process feature selection. After that, we used linear discriminant analysis (LDA) as the classifier. Receiver operating characteristic (ROC) curve analysis was used to evaluate the diagnostic performance of the ADC.

**Results:**

The LDA model based on 13, 8, 3, and 1 features can get the highest area under the ROC curve (AUC) in differentiating BT from MT, PA from WT, PA from MT, and WT from MT on the validation dataset, respectively. Accordingly, the AUC and the accuracy of the model on the testing set achieve 0.7637 and 73.17%, 0.925 and 92.31%, 0.8077 and 75.86%, and 0.5923 and 65.22%, respectively.

**Conclusion:**

The ADC-based radiomics features may be used to assist clinicians for differential diagnosis of PA and WT from MTs.

## Introduction

Salivary gland tumors constitute about 3%–6% of head and neck tumors (1), about 70% of them are located in the parotid gland (2). About 80%–85% of parotid gland tumors are benign tumors (BTs), most of them are pleomorphic adenoma (PA) (about 65% of parotid gland tumors), and Warthin tumor (WT) is the second most common BT (about 15%–20% of parotid tumors) (3). Malignant salivary gland tumors constitute about 15%–30% of parotid gland tumors. Mucoepidermoid carcinoma is the most common parotid gland malignant tumor (MT) (4, 5). About 1.8%–6.2% of PA transforms into MT or carcinoma ex PA, and the recurrence of PA is reported in 0%–3% of patients (6). In contrast, WT rarely undergoes malignant evolution and recurs (7). For the treatment of BT, superficial parotidectomy is preferred, whereas total parotidectomy combined with radiotherapy is preferred for the treatment of MT (2). Specifically, the treatment of PA requires excision by either partial or total parotidectomy, which results in a risk of facial nerve injury (8, 9), the results of the study by Mercante et al. show that total parotidectomy should be the treatment of choice in case of benign parotid gland tumors and in particular for PA (10), whereas the treatment of WT could potentially avoid excision as it can be monitored. Therefore, accurate preoperative diagnosis is essential for treatment.

Fine needle aspiration cytology (FNAC) is a reliable examination that can provide preoperative information about the treatment plan and postoperative procedures (11). As this technology is cheap, fast, safe, and relatively non-invasive, it is commonly used as a mature solution. However, it still suffered from considerable variability in the accuracy, high non-diagnostic rates, and poor sensitivity or specificity (12). When done blindly by clinicians with different levels of experience, poor technique or inaccurate or inadequate sampling can result in a high rate of non-representative or insufficient aspiration (13).

Imaging technology is used to determine the stage of the tumor based on the TNM classification and the suitability of the surgery, which is the main treatment for most parotid gland tumors. Currently, there are a variety of imaging techniques that can be used to study the parotid gland tumors, such as ultrasound, computed tomography (CT), and magnetic resonance imaging (MRI). Ultrasound is an inexpensive and effective tool for delineating cystic or solid tumors, tumor borders, and cervical lymph nodes; however, it has poor visualization of deep lobe and relies on the expertise of the operator (14). CT is not a preferred method for parotid gland tumor evaluation for parotid tumor assessment due to ionizing radiation. MRI plays a crucial role in preoperatively differentiating parotid gland tumors noninvasively (15). The morphological features of parotid gland tumors from conventional MRI can help to distinguish BT and MT (16). Diffusion-weighted imaging (DWI) determines the motion of water molecules qualitatively and translates it into a coefficient called the apparent diffusion coefficient (ADC) (17), which is used to evaluate quantitative water molecule movement through ADC value. DWI is becoming a popular diagnostic and research tool for differential diagnosis of parotid gland tumors. The ADC value of BT is higher than that of MT, and BT is successfully distinguished from MT (16–21). However, previous studies (22–24) reported that ADC value cannot be satisfactorily distinguished between BT and MT, and they did not combine the various ordered imaging features of the whole-tumor region of interest (ROI) with machine learning methods. Even in squamous cell carcinoma of the oral cavity and oropharynx, Bonello et al. did not observe any statistically significant correlation between ADC values and clinical–histological characteristics of SCCA of the oral cavity and oropharynx (25).

Radiomics is one of the most innovative fields of tumor imaging, which involves the use of computer-aided techniques to detect and quantify mathematical patterns in digital images. With the development of artificial intelligence and algorithms, the computer-aided quantitative image evaluation is increasingly applied to improving the accuracy of preoperative diagnosis of parotid gland tumors (26–29), whereas the ADC map–based radiomics in differentiating parotid gland tumors has been addressed in only a few studies and needs further validation (28, 29). The purpose of this study is to evaluate the performance of ADC map–based radiomics analysis with the whole-tumor ROI for differentiating parotid gland (BT vs. MT, PA vs. WT, PA vs. MT, and WT vs. MT).

## Materials and Methods

### Patients

This study was approved by the Ethics Committee of the First Affiliated Hospital of Zhengzhou University (2019-KY-0015-002). The Institutional Review Board waived the requirement of informed consent. All patients’ informed consents were waived for the retrospective nature of this study.

This study retrospectively evaluated the MRI examinations of 130 patients with parotid gland tumors from August 2019 to December 2020. Histopathology diagnosis was obtained in all cases by biopsy or surgical resection. The exclusion criteria were patients (a) with a maximum tumor diameter less than 5 mm, (b) with recurrent tumor, and (c) with poor imaging that was unsuitable for the ROI delineation. A total of 130 patients who underwent a pre-treatment MRI study included 83 men and 47 women, with an average age of 48.22 ± 17.71 years (range 1–85 years). Eighty-eight cases were BT, including 54 (41.54%) PA and 34 (26.15%) WT. The other 42 lesions were MT. The details of patient and tumor characteristics are shown in **Table 1**.

**Table 1:** Distribution of parotid gland tumors.

Characteristic	Number
**Patient**	130
**Age (years) mean ± standard deviation**	48.22 ± 17.71
**Sex (male/female)**	83/47 (63.85%/36.15%)
**Tumor type, n (%)**	130 (100%)
**Benign tumor**	88 (67.69%)
**Pleomorphic adenoma**	54 (41.54%)
**Warthin tumor**	34 (26.15%)
**Malignant tumor**	42 (32.31%)
**Mucoepidermoid carcinoma**	10 (7.69%)
**Salivary duct carcinoma**	4 (3.08%)
**Adenoid cystic carcinoma**	4 (3.08%)
**Squamous cell carcinoma**	4 (3.08%)
**Acinar cell carcinoma**	4 (3.08%)
**Lymphoma**	4 (3.08%)
**Secretory carcinoma**	2 (1.54%)
**Mixed carcinoma**	2 (1.54%)
**Carcinoma in pleomorphic adenoma**	2 (1.54%)
**Myoepithelial carcinoma**	1 (0.77%)
**Epithelial-myoepithelial carcinoma**	1 (0.77%)
**Basal cell carcinoma**	1 (0.77%)
**Adenocarcinoma**	1 (0.77%)
**Rhabdomyosarcoma**	1 (0.77%)
**Sebaceous carcinoma**	1 (0.77%)

### MRI Acquisition Protocols

The MRI data of patients were obtained from the picture archiving and communication system (PACS) of the First Affiliated Hospital of Zhengzhou University. Preoperative plain and contrast-enhanced MRI of the parotid gland was performed for each patient with parotid gland lesion in this study. MRI was performed on three 3.0 T MRI scanners with head/neck coil: a Skyra scanner (Siemens Healthineers, Germany), a Discovery 750 scanner (GE Healthcare, USA), and an Ingenia CX scanner (Philips Healthcare, Holland). The conventional scanning sequences including T1-weighted imaging (T1WI) in axial planes; T2-weighted imaging (T2WI) in axial, sagittal, and coronal planes, axial DWI, and post-contrast (Gadolinium, 0.1 mmol/kg) T1WI in axial, sagittal, and coronal planes were performed. The ADC maps were generated inline after the data acquisition and exported from the PACS workstation to a personal computer in DICOM format (30). A detailed overview of the MRI parameters is listed in **Table 2**. For the MRI data of our 130 patients, cases from Skyra scanner, Discovery 750 scanner, and Ingenia scanner were 92, 26, and 12, respectively.

**Table 2:** MRI main sequence parameters.

Parameters	T2WI	T2WI	T2WI	T1WI	DWI	CE-T1WI	CE-T1WI	CE-T1WI
**Skyra**								
**Imaging technique**	TSE	TSE	TSE	TSE	Readout- segmented EPI	TSE	TSE	TSE
**Orientation**	Coronal	Sagittal	Axial	Axial	Axial	Axial	Sagittal	Coronal
**TR(ms)**	4,500	4,000	4,300	250	3,900	884	884	565
**TE(ms)**	82	82	82	2.5	55	6.9	6.9	6.9
**Field of view (mm^2^)**	230 × 230	230 × 230	230 × 230	230 × 230	220 × 220	240 × 240	240 × 240	240 × 240
**Slice thickness (mm)**	4	4	4	4	4	4	4	4
**No. of slices**	27	25	27	27	24	20	20	20
**b-values (s/mm^2^)**	NA	NA	NA	NA	0/1,000	NA	NA	NA
**Acquisition time**	1 min 13 s	1 min 13 s	1 min 13 s	1 min	1 min 47 s	1 min 37 s	1 min 58 s	59 s
**Discovery 750**								
**Imaging technique**	FSE	FSE	FSE	FSE	EPI	FSE	FSE	FSE
**Orientation**	Coronal Ideal	Sagittal	Axial Ideal	Axial	Axial	Axial	Sagittal Ideal	Coronal
**TR (ms)**	3,410	3,000	2,824	478	3,044.5	550	604	567
**TE (ms)**	68	85	68	Min Full	60.5	Min Full	Min Full	Min Full
**Field of view (mm^2^)**	240 × 240	240 × 240	240 × 240	240 × 240	240 × 240	240 × 240	240 × 240	240 × 240
**Slice thickness (mm)**	4.5	4	4	4	4	4	4	4.5
**No. of slices**	18	22	20	20	20	20	20	20
**b-values (s/mm^2^)**	NA	NA	NA	NA	0/800	NA	NA	NA
**Acquisition time**	1 min 56 s	2 min 2 s	1 min 42 s	38 s	1 min 42 s	1 min 53 s	1 min 56 s	1 min 36 s
**Ingenia CX**								
**Imaging technique**	TSE	TSE	TSE	TSE	EPI	TSE	TSE	TSE
**Orientation**	Coronal	Sagittal	Axial	Axial	Axial	Axial	Sagittal	Coronal
**TR (ms)**	3,400	2,388	3,500	574	3,914	548	486	611
**TE (ms)**	100	66	85	6.5	60	7.1	7.5	7.5
**Field of view (mm^2^)**	180 × 180	220 × 220	180 × 180	180 × 180	200 × 224	200 × 200	180 × 180	200 × 200
**Slice thickness (mm)**	4	4	4	4	4	4	4	4
**No. of slices**	24	24	24	24	24	20	19	24
**b-values (s/mm^2^)**	NA	NA	NA	NA	0/800	NA	NA	NA
**Acquisition time**	1 min 32 s	1 min 33 s	1 min 56 s	1 min 30 s	1 min 38 s	1 min 55 s	2 min 12 s	1 min 43 s

T2WI, T2-weighted imaging; T1WI, T1-weighted imaging; DWI, diffusion-weighted imaging; TSE, turbo spin-echo; EPI, echo-planar imaging; TR, repetition time; TE, echo time; NA, not applicable; FSE, fast spin-echo; CE, contrast enhance.

### ROI Segmentation

**Figure 1:**
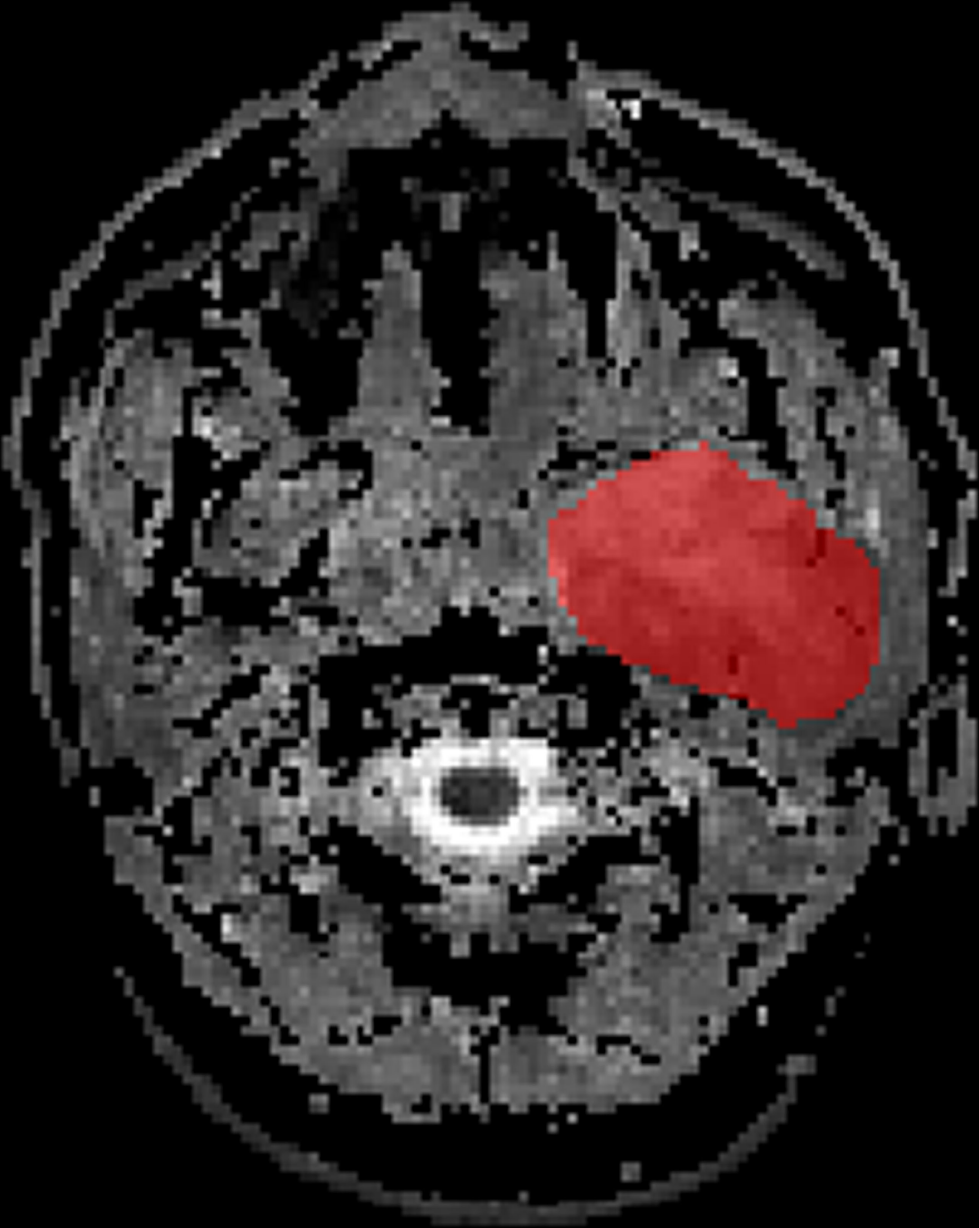
ROI delineation of PA on ADC in ITK SNAP.

The ADC maps were used for our radiomics study. Axial ADC maps were digitally transferred to ITK SNAP (www.itksnap.org). The ROI was manually drawn around the whole tumor margin on each slice of ADC maps (**Figure 1**). All lesions were separately segmented and evaluated by two independent radiologists with 9 and 6 years of experience, respectively, in MRI. The radiologists know nothing about the histological results.

### Feature Extraction

We used the open-source PyRadiomics toolbox to quantify radiomics features from the ADC maps (https://pyradiomics.readthedocs.io/). Three image types (original, wavelet, and gradient) were enabled and then, from each (original and/or derived) image type, extracted the following feature classes: first-order statistics (19 features), gray level co-occurrence matrix (GLCM, 24 features), gray level run length matrix (GLRLM, 16 features), gray level size zone matrix (GLSZM, 16 features), neighboring gray tone difference matrix (NGTDM, 5 features), and gray level dependence matrix (GLDM, 14 features). Meanwhile, 14 shape-based (three-dimensional) features were also extracted from the original image. In total, 944 features were extracted.

To evaluate the relationship between tumor segmentation and extracted imaging features, the intra-class correlation coefficient (ICC) was used to evaluate interobserver reproducibility for the extracted imaging features from the ROI drawn by the two radiologists. The ICC ranged from 0 to 1.00 and was interpreted as follows: r < 0.20, poor; r = 0.20–0.40, fair; r = 0.41–0.60, moderate; r = 0.61–0.75, good; and r > 0.75, excellent. Finally, 260 features were excellent, and the rest 684 features were good.

### Statistical Analysis

In this study, 88 BT (54 PA and 34 WT) and 42 MT of the parotid gland were enrolled. Each case was randomly divided into training and testing cohorts at a ratio of 7:3 and then was compared with each other, respectively, after the following pipeline.

The Synthetic Minority Oversampling TEchnique (SMOTE) was used to remove the unbalance of the training dataset. Then, we applied the normalization process on the feature matrix. For each feature vector, we calculated the mean value and the standard deviation. Each feature vector was subtracted by the mean value and was divided by the standard deviation. After the normalization process, each vector has zero center and unit standard deviation. To reduce the similarity of each feature pair, we calculated the Pearson correlation coefficient (PCC) value of each feature pair and eliminated one of them if the PCC value was larger than 0.95 so that each feature was independent to each other. Then, we used recursive feature elimination (RFE) algorithm to process feature selection, which is based on a classifier that recursively considers smaller set of features in the training dataset by ranking features by importance until the specified number of features remains.

We used linear discriminant analysis (LDA) as the classifier. LDA was a linear classifier by fitting class conditional densities to the data and using Bayes’ rule. To determine the hyper-parameter (e.g., the number of features) of a model, we applied cross-validation with five-fold on the training dataset. The hyper-parameters were set according to the model performance on the validation dataset.

Receiver operating characteristic (ROC) curve analysis was used to evaluate the diagnostic performance of the ADC map–based radiomics features for differential diagnosis of parotid gland tumors (BT and MT, PA and WT, PA and MT, and WT and MT). The area under the ROC curve (AUC) was calculated for quantification. The accuracy, sensitivity, specificity, positive predictive value (PPV), and negative predictive value (NPV) were also calculated at a cutoff value that maximized the value of the Youden index. We also estimated the 95% confidence interval by bootstrap with 1,000 samples. All the above processes were implemented with FeAture Explorer Pro (FAEPro, version 0.4.0) on Python (3.7.6).

## Results

### BT (PA + WT) vs. MT

**Figure 2:**
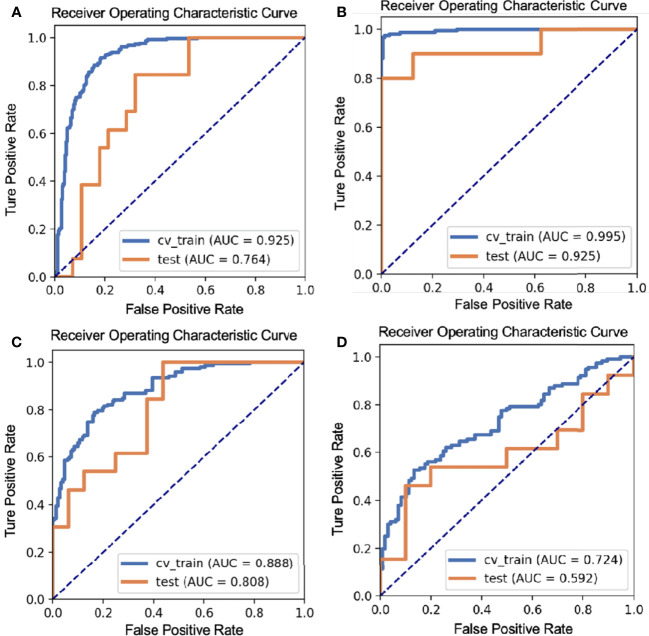
The ROC curves of different parotid gland tumors: **(A)** BT vs. MT; **(B)** PA vs. WT; **(C)** PA vs. MT; **(D)** WT vs. MT.

The LDA model based on eight features can get the best diagnostic performance on the testing set in differentiating BT (PA + WT) from MT. The AUC and the accuracy could achieve 0.7637 and 73.17%, yielding sensitivity and specificity 84.62% and 67.86%, respectively. The diagnostic performance of significant ADC radiomics parameters and the selected features in differentiating PA from MT were shown in **Tables 3**, **4**. The ROC curve was shown in **Figure 2A**.

**Table 3:** ROC analysis of ADC radiomics parameters.

Statistics	Value
**Accuracy**	0.7317
**AUC**	0.7637
**AUC 95% CIs**	[0.6179–0.9106]
**NPV**	0.9048
**PPV**	0.5500
**Sensitivity**	0.8462
**Specificity**	0.6786
**Youden Index**	0.3673

**Table 4:** The coefficients of features in the model.

Features	Coef in Model
**Original shape sphericity**	−1.436
**Wavelet-LHL first-order mean**	1.223
**Wavelet-LHH gldm large dependence low–gray level emphasis**	−1.313
**Wavelet-HHL first-order mean**	−0.423
**Wavelet-HHL glszm small-area low–gray level emphasis**	1.909
**Wavelet-LLL glszm small-area low–gray level emphasis**	0.230
**Gradient glcm cluster tendency**	0.885
**Original glszm small-area low–gray level emphasis**	−1.854

### PA vs. WT

The LDA model based on 13 features can get the best diagnostic performance on the testing set in differentiating PA from WT. The AUC and the accuracy could achieve 0.925 and 92.31%, yielding sensitivity and specificity 80.00% and 100.00%, respectively. The diagnostic performance of significant ADC radiomics parameters and the selected features in differentiating PA from WT were shown in **Tables 5**, **6**. The ROC curve was shown in **Figure 2B**.

**Table 5:** ROC analysis of ADC radiomics parameters.

Statistics	Value
**Accuracy**	0.9231
**AUC**	0.9250
**AUC 95% CIs**	[0.7778–1.0000]
**NPV**	0.8889
**PPV**	1.0000
**Sensitivity**	0.8000
**Specificity**	1.0000
**Youden Index**	0.9554

**Table 6:** The coefficients of features in the model.

Features	Coef in Model
**Wavelet-LHL glrlm gray level non-uniformity normalized**	−2.398
**Wavelet-LHL glszm small-area low–gray level emphasis**	1.179
**Wavelet-HLL first-order median**	1.300
**Wavelet-HLH glszm gray level non-uniformity normalized**	0.095
**Wavelet-HLH glszm gray level variance**	−3.308
**Wavelet-HHH glcm Imc1**	−1.490
**Gradient ngtdm complexity**	0.115
**Original first-order 10 percentile**	−4.296
**Original first-order median**	2.820
**Original first-order skewness**	1.726
**Original gldm large dependence high gray level emphasis**	0.929
**Original glcm autocorrelation**	−5.527
**Original glcm joint average**	4.310

### PA vs. MT

The LDA model based on three features can get the best diagnostic performance on the testing set in differentiating PA from MT. The AUC, the accuracy, sensitivity, and specificity could achieve 0.8077, 75.86%, 100.00%, and 56.25%, respectively. The diagnostic performance of significant ADC radiomics parameters and the selected features in differentiating PA from MT were shown in **Tables 7**, **8**. The ROC curve was shown in **Figure 2C**.

**Table 7:** ROC analysis of ADC radiomics parameters.

Statistics	Value
**Accuracy**	0.7586
**AUC**	0.8077
**AUC 95% CIs**	[0.6381–0.9474]
**NPV**	1.0000
**PPV**	0.6500
**Sensitivity**	1.0000
**SpecificityYouden Index**	0.56250.4557

**Table 8:** The coefficients of features in the model.

Features	Coef in Model
**Wavelet-LLH first-order 90 percentile**	0.684
**Wavelet-LHH gldm large dependence low–gray level emphasis**	−0.497
**Original first-order 10 percentile**	−1.468

### WT vs. MT

The LDA model based on one feature can get the highest AUC on the testing set in differentiating WT from MT. The AUC, accuracy, sensitivity, and specificity could achieve 0.5923, 65.22%, 46.15%, and 90.00%, respectively. The diagnostic performance of significant ADC radiomics parameters and the selected features in differentiating WT from MT was shown in **Tables 9**, **10**. The ROC curve was shown in **Figure 2D**.

**Table 9:** ROC analysis of ADC radiomics parameters.

Statistics	Value
**Accuracy**	0.6522
**AUC**	0.5923
**AUC 95% CIs**	[0.3413–0.8250]
**NPV**	0.5625
**PPV**	0.8571
**Sensitivity**	0.4615
**SpecificityYouden Index**	0.90000.6549

**Table 10:** The coefficients of features in the model.

Features	Coef in Model
**Wavelet-LHH glcm Imc1**	0.763

## Discussion

Using ADC-based radiomics analysis to detect parotid gland tumors has increasingly shown its value on different histopathological entities (30–33). ADC-based texture analysis, as a non-invasive and quantitative additional supporting tool, can extract features of entire tumors and go beyond individual-based visual assessment (34). Previously, many studies (26, 34–42) have explored the computer-assisted discrimination of benign and malignant parotid gland tumors, but only a few studies have evaluated the role of ADC-based radiomics features in the differentiation of parotid lesions (29, 31, 34). In our study, the ADC-based radiomics features were from three different manufacturers, which still shows a good performance in differentiating PA and WT from MTs. This implies the advantage of the generalization of our ADC-based features that can cross different manufacturers.

It was observed that intra/inter-tumoral heterogeneity and overlap of ADC values between BT and MT could be overcome by making a whole-tumor analysis (30, 31, 43). Previously, Ma et al. reported that no significant difference between BT and MT was found in ADC histogram parameters extracted from the ROI of whole-tumor ADC map (30). We used LDA as the classifier and found a significant difference in ADC map–based radiomics features between parotid gland BT and MT. The AUC of this model is 0.7637 in sensitivity of 84.62% and specificity of 67.86%, which may be explained by that WT was not the dominant tumor among BT. The specificity of our result is slightly lower, this could arise from that both parotid gland BT and MT have been well-differentiated and exhibiting cytological overlap.

In the comparison of PA and WT, we found extracted radiomics features get excellent diagnostic performance; the AUC is 0.925 in sensitivity of 80.00% and specificity of 100.00%; these results agree with findings of previous reports (28, 30, 31), and it may be due to the tumor components. PA exhibits a variety of histopathologic characteristics, and the presence of epithelial, mesenchymal-like tissues and rich mucus is the main diagnostic feature, which leads to facilitated water diffusibility and the highest ADC values (18, 44, 45). However, WT has lymphoid stroma with low ADC values (17, 46).

We found that the AUC was 0.8077 in sensitivity of 100.00% and specificity of 56.25% in distinguishing PA from MT, and the diagnostic performance of ADC map–based radiomics features was high, which agreed with findings of previous reports (28, 30, 31). Heterogeneous ADC maps were seen in MT (47). Investigators reported that myxoid lymphosarcomas, adenoid cystic carcinomas, and mucoepidermoid carcinomas had higher ADC values compared with other malignant neoplasms, whereas lymphomas had lower ADC values (17), but ADC values of MT were lower than these of PA.

Comparing WT with MT, our study showed that the AUC was 0.592 in sensitivity of 46.15% and specificity of 90.00%, and the diagnostic performance of ADC map–based radiomics features was not high, which is due to WT having high cellularity. The histologic structure of WT that can resemble MT includes both an oncocytic epithelial component and lymphoid stroma (17, 46, 48).

This study has some limitations. First, the data were performed on three 3.0 T MRI scanners, and different parameters might affect the diagnostic performances of the ADC map–based radiomics features; MRI acquisition parameters certainly need to be considered in the further clinical application of this technology. Second, we only constructed radiologic features based on ADC maps, and combining T2WI and contrast-enhanced T1WI is needed to accumulate more evidence for future clinical applications. Third, it is a retrospective study with relatively fewer cases, especially some pathological categories in MT. They included only PA and WT as BTs and that the dataset was unbalanced (BT was more represented than MT). We will continue to collect cases and expand the sample size. Last but not least, we did not validate our model with an external dataset. Multicenter studies with a larger number of patients are needed to further research.

## Conclusion

In this study, we proposed to use ADC-based radiomics features for differential diagnosis of PA and WT from MT, which shows very good predictive performance. This implies that the radiomics analysis can be used as an additional tool for supporting radiologists’ decisions. Further validation in a larger prospective study is required for this method.

## Data Availability Statement

The original contributions presented in the study are included in the article/supplementary material. Further inquiries can be directed to the corresponding author.

## Ethics Statement

This study was conducted with approval from the Review Committee of the First Affiliated Hospital of Zhengzhou University (No: 2019-KY-0015-002). All patients’ informed consents were waived for the retrospective nature of this study. Written informed consent from the participants’ legal guardian/next of kin was not required to participate in this study in accordance with the national legislation and the institutional requirements.

## Author Contributions

BW and HH designed this study. BW, ZZ, and YL acquired data. BW and ZZ drafted the manuscript. HH, JZ, and LL performed the data and statistical analysis. JC and YZ contributed substantially to its revision. All authors contributed to the article and approved the submitted version.

## Funding

This work was supported by the Fund of Henan Medical Science and Technology Research Plan (LHGJ20190157).

## Conflict of Interest

Author HH was employed by Philips Healthcare.

The remaining authors declare that the research was conducted in the absence of any commercial or financial relationships that could be construed as a potential conflict of interest.

## Publisher’s Note

All claims expressed in this article are solely those of the authors and do not necessarily represent those of their affiliated organizations, or those of the publisher, the editors and the reviewers. Any product that may be evaluated in this article, or claim that may be made by its manufacturer, is not guaranteed or endorsed by the publisher.
